# A pan-cancer analysis of the expression and molecular mechanism of DHX9 in human cancers

**DOI:** 10.3389/fphar.2023.1153067

**Published:** 2023-05-04

**Authors:** Yanfeng Wang, Yongxin Guo, Yanping Song, Wenbo Zou, Junjie Zhang, Qiong Yi, Yujie Xiao, Jing Peng, Yingqi Li, Lei Yao

**Affiliations:** ^1^ Department of Anesthesiology, Xiangya Hospital, Central South University, Changsha, Hunan, China; ^2^ Anesthesia and Operation Center, The First Medical Center of Chinese PLA General Hospital, Beijing, China; ^3^ Department of Anesthesiology, No. 922 Hospital of PLA, Hengyang, Hunan, China; ^4^ Department of General Surgery, No. 924 Hospital of PLA Joint Logistic Support Force, Guilin, Guangxi, China; ^5^ Department of General Surgery, Xiangya Hospital, Central South University, Changsha, Hunan, China; ^6^ National Clinical Research Center for Geriatric Disorders (Xiangya Hospital), Changsha, China

**Keywords:** DHX9, cancer, prognosis, phosphorylation, immunotherapy

## Abstract

Finding new targets is necessary for understanding tumorigenesis and developing cancer therapeutics. DExH-box helicase 9 (DHX9) plays a central role in many cellular processes but its expression pattern and prognostic value in most types of cancer remain unclear. In this study, we extracted pan-cancer data from TCGA and GEO databases to explore the prognostic and immunological role of DHX9. The expression levels of DHX9 were then verified in tumor specimens by western blot and immunohistochemistry (IHC). The oncogenic roles of DHX9 in cancers were further verified by *in vitro* experiments. We first verified that DHX9 is highly expressed in most tumors but significantly decreased in kidney and thyroid cancers, and it is prominently correlated with the prognosis of patients with different tumors. The phosphorylation level of DHX9 was also increased in cancers. Enrichment analysis revealed that DHX9 was involved in Spliceosome, RNA transport and mRNA surveillance pathway. Furthermore, DHX9 expression exhibited strong correlations with immune cell infiltration, immune checkpoint genes, and tumor mutational burden (TMB)/microsatellite instability (MSI). In liver, lung, breast and renal cancer cells, the knockdown or depletion of DHX9 significantly affected the proliferation, metastasis and EMT process of cancer cells. In summary, this pan-cancer investigation provides a comprehensive understanding of the prognostic and immunological role of DHX9 in human cancers, and experiments indicated that DHX9 was a potential target for cancer treatment.

## Introduction

Tumorigenesis is a complex process that may be accompanied by oncogene activation or tumor suppressor gene inactivation (Chaffer and Weinberg, 2015). Metastasis and tumor recurrence often lead to account treatment failure and poor prognosis (Ganesh and Massague, 2021). It is necessary to reveal the mechanisms of tumorigenesis, identify effective biomarkers and develop novel therapies to improve patients’ outcomes (Hao and Li, 2020). DExH-Box helicase 9 (DHX9), also known as RNA helicase A (RHA), is a kind of nucleic acid unwinding enzyme that can unwind DNA and RNA double-stranded and other complex polynucleotide structures (Chakraborty and Grosse, 2011). DHX9 plays a central role in many cellular processes including regulation of DNA replication, transcription, RNA transport, translation, microRNA and circular RNA processing, and genome maintenance (Chakraborty and Grosse, 2010; Jain et al., 2013; Ding et al., 2019). Besides, DHX9 is a multi-domain and multi-functional enzyme, when it is deregulated, it can alter cellular growth and result in tumor formation (Jain et al., 2013; Cristini et al., 2018). Therefore, DHX9 may be a new target for the treatment of malignant tumors.

As tumor cells interact with infiltrating immune cells in tumor microenvironment (TME), they contribute to tumor occurrence and development (Bindea et al., 2013; Muenst et al., 2016). Immunotherapy targeting their interaction has become a new hope for antitumor therapy in recent years, especially the blockade of immune checkpoints (Pardoll, 2012). Although the immune-checkpoint inhibitors have been approved to treat a wide range of malignancies, such as target cytotoxic T lymphocyte protein 4 (CTLA-4) or the programmed cell death protein 1 (PD-1)–programmed cell death 1 ligand 1 (PD-L1) axis (Topalian et al., 2012; Routy et al., 2018), only a limited percentage of patients respond well (Topalian et al., 2015). It is necessary to explore other potential targets. DHX9 also has an effect in the field of innate immunity. DHX9 knockout was reported may lead to senescence in primary human diploid fibroblasts, which is an important mechanism for antitumor responses (Luo et al., 2013; Lee et al., 2014). However, the correlation between DHX9 TME in cancers remains elusive.

This study integrated the pan-cancer analysis and *in vitro* and vivo experiments to characterize the role of DHX9 in various cancers, which would provide a better understanding of tumorigenesis and progression, and help to discover novel targets for cancer treatment.

## Materials and methods

### Gene expression and protein phosphorylation analysis

The TIMER2 (Tumor Immune Estimation Resource, Version 2) tool’s “Gene_DE” module was used to compare DHX9 expression variations between different cancers and these respective normal tissues. For cancers lacking comparable normal tissues, the “Expression Analysis-box Plots” module in the GEPIA2 (Gene Expression Profiling Interactive Analysis, Version 2) tool implemented within the GTEx (Genotype-Tissue Expression) database. In addition, we used the “Pathological Stage Plot” module of GEPIA2 to obtained the violin plots of DHX9 expression in all TCGA tumors at different pathological stages. Oncomine database was used to screen≧ 7 eligible studies by setting corresponding screening conditions (*p*-value <0.05, fold change> 2, and gene rank: top 10%).

We used the UALCAN portal to analyze protein expression and phosphorylation level of DHX9 in CPTAC (Clinical Proteomic Tumor Analysis Consortium) datasets, which included breast cancer, ovarian cancer, colon cancer, clear cell RCC (ccRCC, renal cell carcinoma), UCEC (uterine corpus endometrial carcinoma), and LUAD (lung adenocarcinoma).

### Survival analysis

The OS (overall survival) and DFS (disease-free survival) significance of DHX9 among TCGA tumors was obtained from GEPIA2 with a median cut-off value. The log-rank test was used for estimate the prognostic significance of DHX9. Kaplan-Meier plotter was used for visualization of Kaplan-Meier plot in breast cancer, ovarian cancer, lung cancer, gastric cancer, and liver cancer.

### Enrichment analysis of DHX9-related genes

The fifty DHX9-binding proteins validated by experiments were obtained from the Search Tool for the Retrieval of Interacting Genes/Proteins (STRING) website. Next, the top 100 similar genes of DHX9 were extracted from GEPIA2. Pearson correlation was used to identify 8 DHX9-related genes via GEPIA2. The expression of 8 selected genes in TCGA tumors was visualized by TIMER2.

We used Venn to analyze the intersection of DHX9 binding protein and related genes. These intersected genes were subjected to David website for KEGG (Kyoto Encyclopedia of Genes and Genomes) pathway analysis. Furthermore, the R software package " clusterprofiler” was used to conduct GO (gene ontology) enrichment analysis.

### Immune infiltration and immunotherapy analysis

Assessment of immune infiltration was carried out using the TIMER and XCELL algorithms. To analyze the correlation between DHX9 expression and immune checkpoint molecules in TGCA cancers, Spearman correlation analysis was conducted, and the outcomes were visualized by a heatmap and scatter plot.

As for TMB (tumor mutational burden)/MSI (microsatellite instability) analysis, we used the sangerbox tool (Bonneville et al., 2017) to explore the potential correlation between DHX9 expression and TMB/MSI in different tumors.

### Tumor specimens and immunohistochemistry (IHC)

All tissue specimens involved in this study were freshly obtained from patients undergoing surgical treatment at Xiangya Hospital, Central South University. Ethical approval was approved by the ethics committee of Xiangya Hospital, Central South University.

Tumor tissue sections were processed according to standard protocols. After heated and dewaxed with an alcohol gradient treatment, slides were blocked with 3% normal sheep serum (ZSbio, Beijing, China) for 1 h at room temperature. Then, specimens will be incubated overnight at 4°C with primary antibody (DHX9, 1:100; TGFβR1, 1:100). The VECTASTAIN Elite ABC HRP kit (Vector Laboratories, Burlingame, CA, United States) and the VECTOR DAB kit (Vector Laboratories) were then used to color development according to the manufacturer’s instructions. The slides were visualized using Pannoramic Digital Slide Scanners (3DHISTECH, Budapest, Hungary).

### Cell lines, RNA interfering and western boltting

Liver cancer cells Hep-3B and SK-Hep-1 were purchased from the Chinese Academy of Science Cell Bank (Shanghai, China). ccRCC cells ACHN and OS-RC-2 were purchased form Icellbioscience (Shanghai, China). Thoracic cancer cells H1299 and PC9 were kindly provided by Professor Ruimin Chang (Department of Thoracic Surgery, Xiangya Hospital, Central South University), and breast cancer cells MDA-MB-231 and MCF7 were kindly provided by Professor Yuhui Wu (Department of General Surgery, Xiangya Hospital, Central South University). All cells were cultured in high-glucose DMEM medium (Hyclon, Logan, UT, United States) supplemented with 10% fetal bovine serum (164210–500, ProCell, Wuhan, China), 100 U/mL penicillin, and 100 μg/mL streptomycin (15070063, Gibco) and incubated at 37°C and 5% CO_2_.

The DHX9 specific Small Interfering RNA (siRNA) molecules were synthesized by GenePharma (Shanghai, China). The specific sequences of these siRNAs are as follows: si-DHX9-1: 5′—GGU​GCC​GCU​UGC​AGA​CAU​UTT AAU​GUC​UGC​AAG​CGG​CAC​CTT--3’; si-DHX-2: 5’--GGG​CAG​CAA​CUA​CCU​GAU​UTT AAU​CAG​GUA​GUU​GCU​GCC​CTT--3’. All siRNA transfection experiments were performed using Lipofectamine-3000 (L3000015, Invitrogen, Eugene, OR, United States) according to the manufacturer’s protocols. The protein expression level of DHX9, TGFβR1 and epithelial-mesenchymal transition (EMT) relative biomarkers in different cancer cell lines were detected by western blotting. Western blotting assay was implemented as described previously (Yao et al., 2022). The primary antibodies: DHX9 (#15309-1-AP, Proteintech, 1:1,000), E-Cadherin (CST #14472, 1:1000), ZO-1 (CST #13663, 1:1000), *ß*-Catenin (CST #8480, 1:1000), N-Cadherin (Abcam, ab18203, 1:1000), Vimentin (CST #5741, 1:1000), *a*-SMA (CST #19245, 1:1000), TGFβR1 (ZENBIO, 346599, 1:1000), *ß*-Tubulin (Servicebio, GB11017, 1:2000).

### Invasion, wound healing and colony formation assay

Transwell cell invasion assay, wound healing assay and colony formation assay were performed as previously described (Yao et al., 2022).

### Immunofluorescence

Cells were inoculated with 24-well plates and cultured overnight, then fixed with formaldehyde and permeabilized with 0.1% Triton X-100. Blocking cells with 3% normal sheep serum (ZSbio, Beijing, China), then cells will be incubated with primary antibodies (DHX9, 1:200; TGFβR1, 1:200) overnight at 4°C. Next day, after incubated the fluorophore-conjugated secondary antibody, cell nuclei were be labelled by Fluoroshield mounting medium with DAPI (Abcam). Fluorescence images are acquired by a multi-channel fluorescence microscope (Nikon, Japan).

### Generation of DHX9-deficient cells via CRISPR/Cas9 editing

The knockout of DHX9 was attempted by means of designed guide RNAs (sgRNAs, AGG​UUA​UAA​UUA​CAC​UGG​CA) by the Synthego online tool (https://design.synthego.com/#/). A DNA fragment was created by annealing the forward primer CAC​CGA​GGT​TAT​AAT​TAC​ACT​GGC​A and reverse primer AAA​CTG​CCA​GTG​TAA​TTA​TAA​CCT​C with respect to the sgRNA sequence. The DNA fragment was further modified through the integration into pSpCas9(BB)-2A-GFP (PX458) (Addgene plasmid ID: 48138), following established protocols (Ran et al., 2013). SK-Hep-1, PC9, MCF7and OS-RC-2 cells were transfected with sgRNA plasmid and cells with green fluorescent were collected via flow cytometry. DHX9 knockout was ultimately verified by western blotting.

### *In vivo* xenografts

Female BALB/c nude mice at 4–6 weeks of age, purchased from Hunan SJA Laboratory Animal Co. Ltd. (Changsha, Hunan, China) were used in this study. Subcutaneous xenograft was established via injecting of 100 μL PBS containing 5 × 10^6^ wild-type or DHX9-KO Hep-3B cells. Animal experiments were approved by the Animal Ethics Committee of Hunan SJA Laboratory Animal Co. Ltd. and performed following Guidelines for the Care and Use of Laboratory Animals at Central South University.

### Statistical analysis

All statistical analyses were performed using Prism (Version 9.0) software and R language software version R-4.0.3. The statistical significance was determined using the 0.05 *p*-value, and all *p*-values were two-tailed.

## Results

### DHX9 expression in pan-cancer

DHX9 (NM_001357.5 for mRNA or NP_001348.2 for protein, Supplementary Figure S1A) is located in chromosome 1. Its structure is usually composed of DSRM (cl00054) domain, HELICc (cd00079) domain, and OB_NTP_bind (pfam07717) domain (Supplementary Figure S1B). Phylogenetic tree data revealed that DHX9 has evolved from nematodes to primates among different species (Supplementary Figure S1C). DHX9 was widely expressed in different tissues with low RNA tissue specificity (Supplementary Figures S1D-E).

**FIGURE 1:**
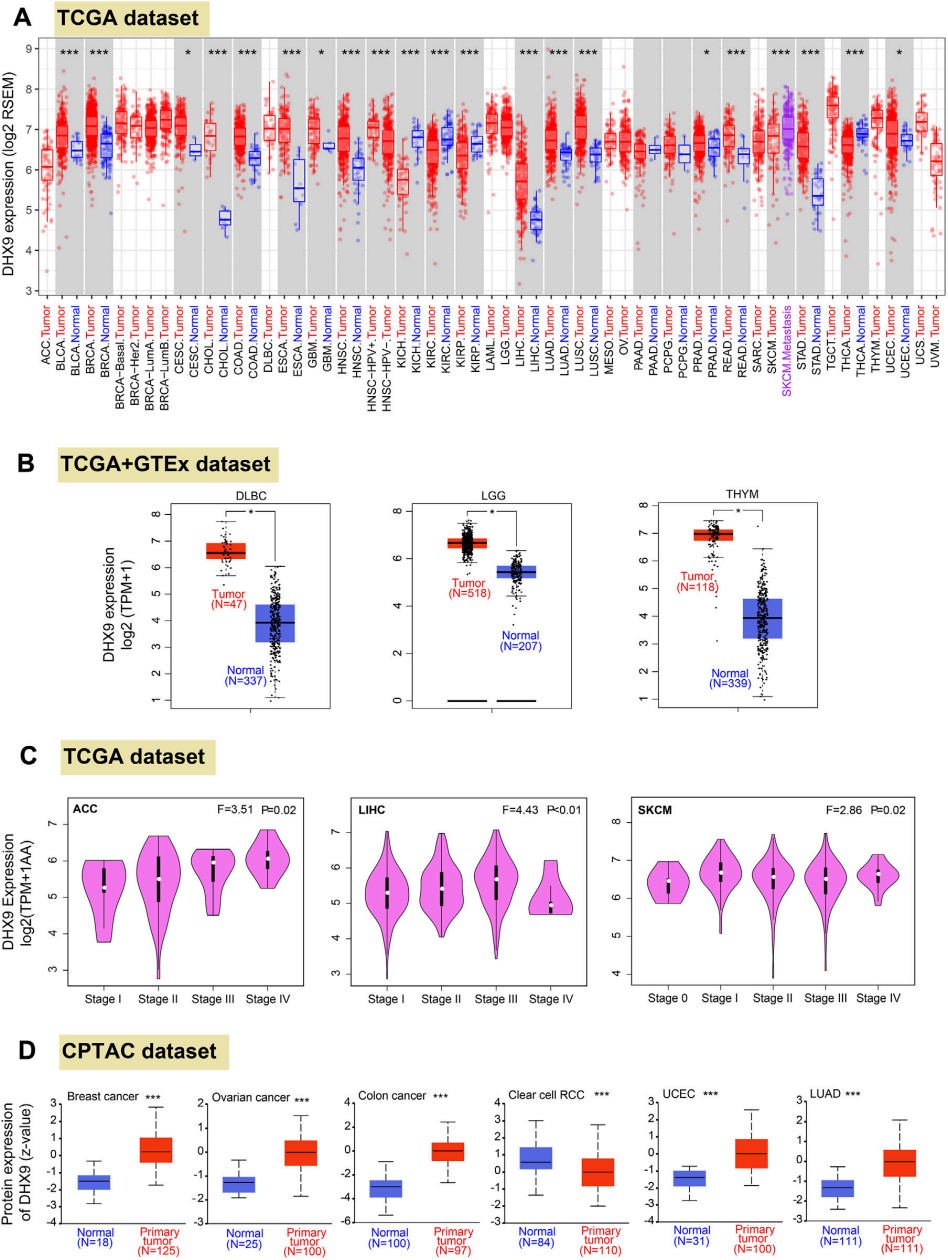
Differential expression patteerns of DHX9 across various cancers and pathological stages. **(A)** Based on the TCGA dataset, the DHX9 expression in different cancers through TIMER2. **(B)** For the type of DLBC, LGG, and THYM in the TCGA project, we used the GTEx database to provide the corresponding normal tissues as controls. The box plot data of comparison between tumor and normal tissues were supplied. **(C)** The DHX9 expression in different pathological stages of ACC, LIHC and SKCM. **(D)** The protein expression levels of DHX9 between tumor and normal tissues of breast cancer, ovarian cancer, colon cancer, ccRCC, UCEC, and LUAD. * *p* < 0.05; ** *p* < 0.01; *** *p* < 0.001.

Firstly, we profiled the pan cancer expression pattern of DHX9 based on the TGCA database. In 33 cancers, DHX9 was highly expressed in BLCA (bladder urothelial carcinoma), BRCA (breast invasive carcinoma), CHOL (cholangiocarcinoma), COAD (colon adenocarcinoma), ESCA (esophageal carcinoma), HNSC (head and neck squamous cell carcinoma), LIHC (liver hepatocellular carcinoma), LUAD, LUSC (lung squamous cell carcinoma), READ (rectum adenocarcinoma), STAD (stomach adenocarcinoma) and CESC (cervical squamous cell carcinoma and endocervical adenocarcinoma), GBM (glioblastoma multiforme), PRAD (prostate adenocarcinoma), and UCEC (Figure 1A). However, DHX9 expression in tumor tissue was significantly lower than that in the normal control group in KICH (kidney chromophobe), KIRC (kidney renal clear cell carcinoma), KIRP (kidney renal papillary cell carcinoma), THCA (thyroid carcinoma) (Figure 1A).

Since the TCGA database could not provide the normal tissues of some tumors for comparison, we extracted expression data from GTEx and found that DHX9 was highly expressed in DLBC (lymphoid neoplasm diffuse large B-cell lymphoma), LGG (brain lower grade glioma), THYM (thymoma) compared with normal tissues (Figure 1B), whereas no significant differences were found from normal tissue and tumor tissues in other tumors (Supplementary Figure S2A).

In Oncomine database, DHX9 expression was significantly increased in 51 items and significantly decreased in 9 items (Supplementary Figure S3). It was further confirmed that DHX9 was highly expressed in breast cancer, lung cancer, sarcoma, colorectal cancer and brain tumor (Supplementary Figures S3A-E). We also used the GEPIA2 database to analyze the correlation between DHX9 expression and clinicopathological stages of various cancers and found there was a statistical difference in ACC, LIHC and SKCM (Figure 1C), while most other cancers showed no statistical difference (Supplementary Figures S2B-E). As for protein expression levels, CPTAC database results showed that DHX9 total protein expression in breast cancer, ovarian cancer, colon cancer, UCEC, and LUAD tissues was higher than that in normal tissues, but the expression of ccRCC was lower than that of normal tissues (Figure 1D).

In our study, both upregulated mRNA and protein expressions of DHX9 were detected in BRCA, COAD, LUAD, and UCEC, while both downregulation of mRNA and protein expression could only be detected in KIRC, which was further validated by IHC (Supplementary Figures S4A-D) and UCEC was exempt because of absent normal tissue.

### Prognostic value of DHX9 in pan-cancer

**FIGURE 2:**
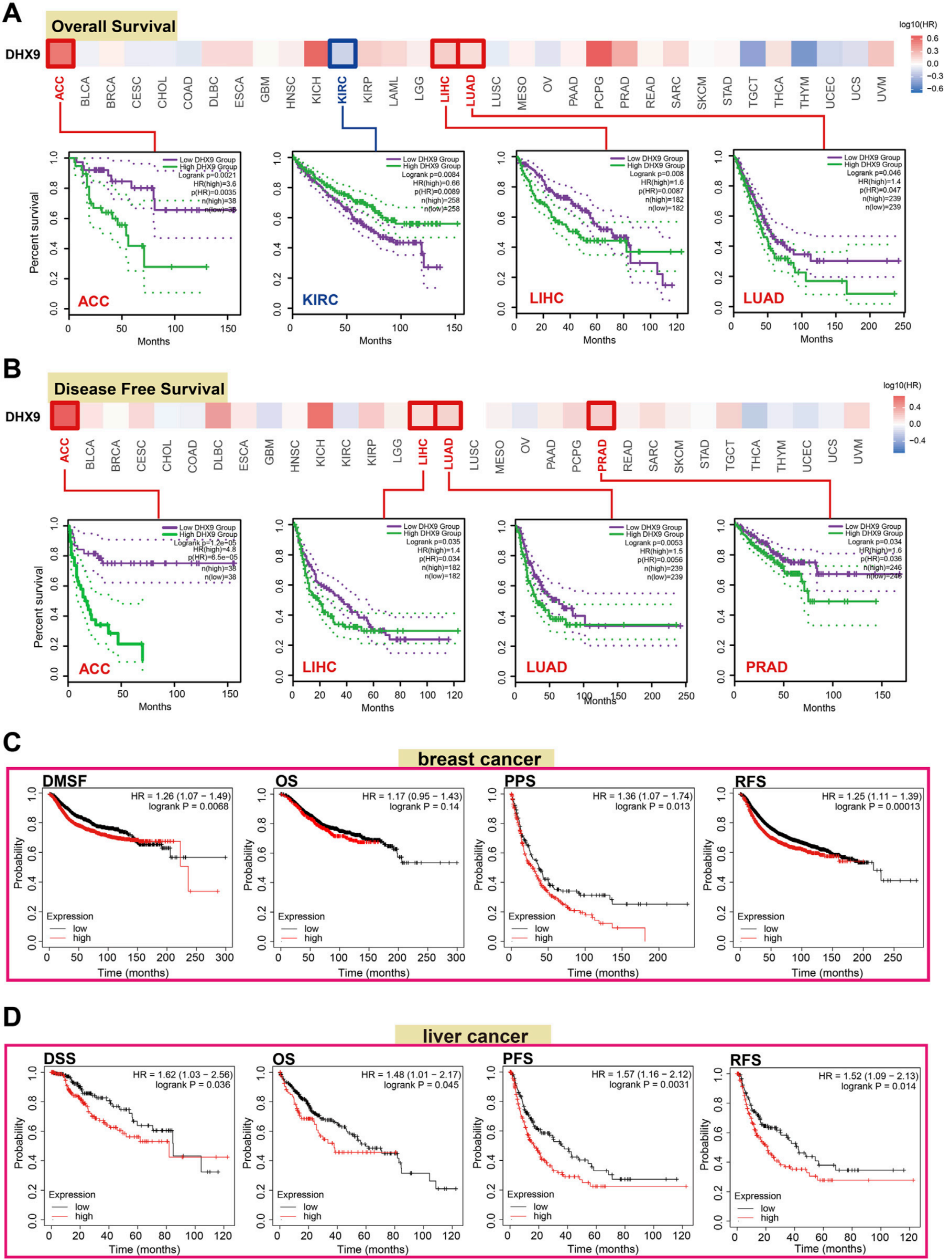
The role of DHX9 in cancer survival prognosis in TCGA. **(A, B)** The OS and DFS data of DHX9 in different cancers. **(C, D)** The Kaplan-Meier plotter was used to display the survival prognosis of the DHX9 in breast cancer and liver cancer cases.

The prognostic value is a key property of an oncogene, so we investigated the prognostic value of DHX9 in cancers. In TCGA, the high expression of DHX9 was associated with poor prognosis of OS in ACC, LIHC and LUAD, whereas its low expression was related to poor OS in KIRC (Figure 2A). In addition, high expression of DHX9 was significantly correlated with poor DFS in ACC, LIHC, LUAD, and PRAD (Figure 2B). Furthermore, high expression of DHX9 was significantly associated with poor RFS (relapse-free survival), DMFS (distant metastasis-free survival), and PPS (post-progression survival) in patients with breast cancer (Figure 2C). In liver cancer patients, a elevated expression of DHX9 was found to be significantly associated with an unfavorable OS, RFS, PFS, and disease-specific survival (DSS) (Figure 2D). These results indicated that DHX9 was a prognostic biomarker for cancer and especially critical for breast cancer and liver cancer.

### Pan-cancer analysis of the DHX9 phosphorylation

**FIGURE 3:**
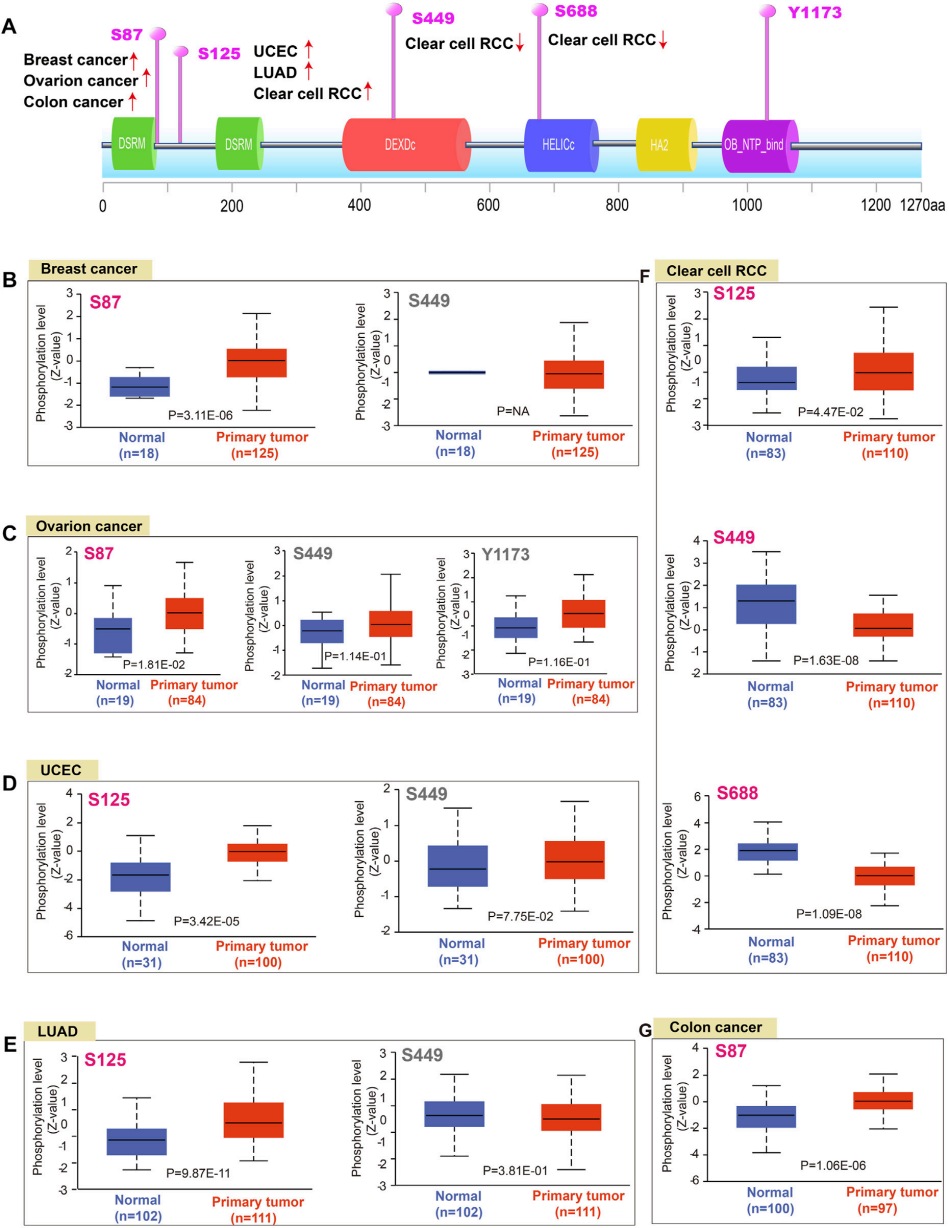
Phosphorylation analysis of DHX9 protein in different tumors. **(A)** The schematic diagram and positive phosphoprotein sites of DHX9 protein (NP_001348.2, S87, S125, S449, S688, and Y1173 sites). **(B–G)** The phosphorylation levels between normal and tumor tissues were analyzed by UALCAN.

DNA phosphorylation is a regulatory mechanism of gene expression through modulating gene readability. Herein, we found that the S87 site of DHX9 between the two DSRM domains of DHX9 demonstrated a higher phosphorylation level in breast cancer, ovarian cancer and colon cancer than those in normal tissues (Figures 3A, B). Then, the phosphorylation level of S125 locus between the two DSRM domains was higher in UCEC, LUAD and ccRCC than that of normal tissue (Figures 3D–F). The S449 site in the DEXDc domain expressed in breast cancer, UCEC, LUAD, ovarian cancer, and ccRCC, but there was a significant difference of lower phosphorylation only in ccRCC compared with normal tissues (Figures 3B–F). The S688 site was located in the HELICc domain, and its phosphorylation level was only observed in the ccRCC, which was lower than normal tissues (Figure 3F). In contrast, site Y1173 located in the OB_NTP_bind domain showed phosphorylation changes only in ovarian cancer with no significant difference (Figure 3C).

### Enrichment analysis of DHX9-related partners

**FIGURE 4:**
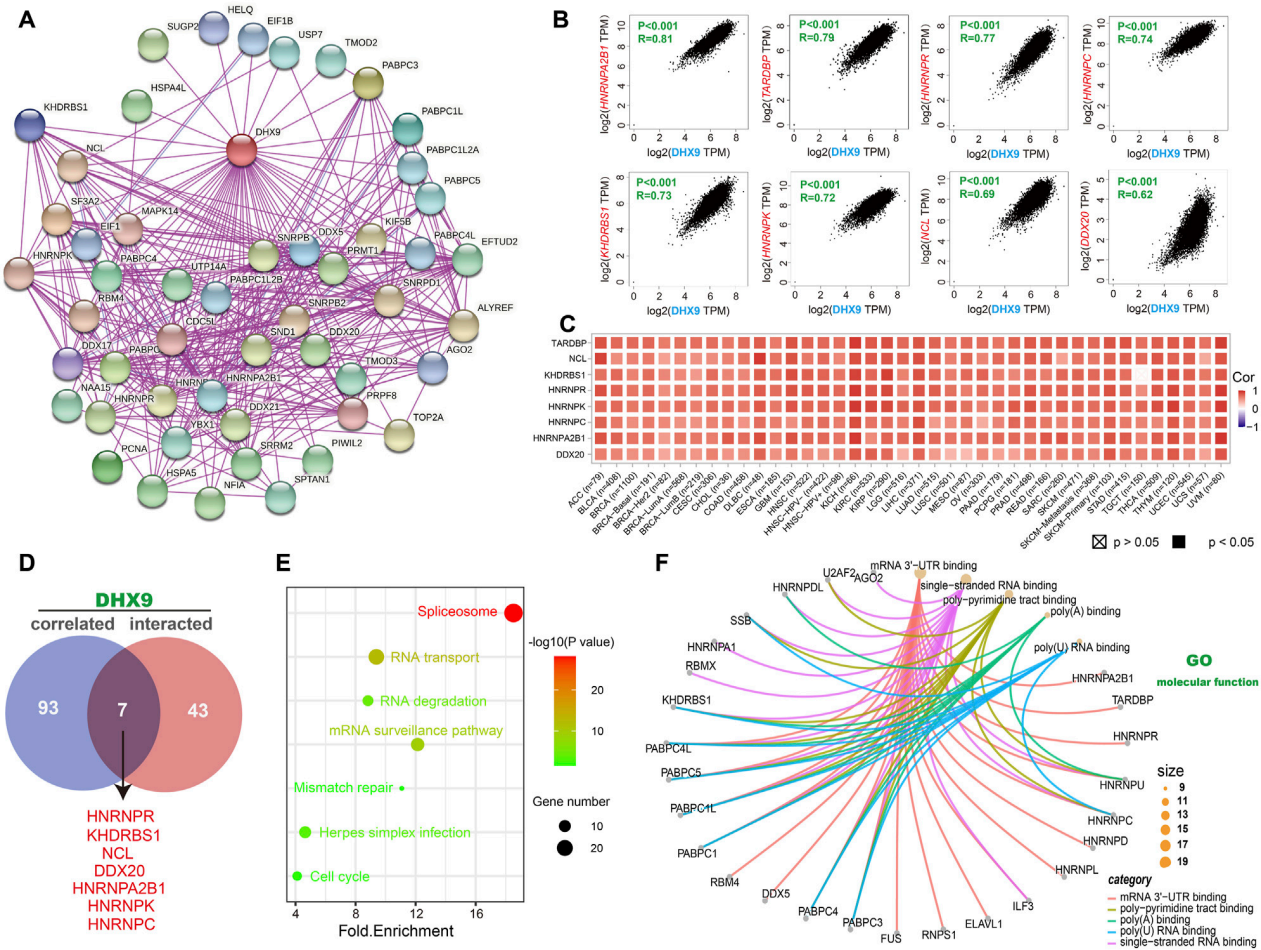
DHX9-related gene enrichment analysis. **(A)** Experimentally confirmed DHX9-binding proteins were obtained through the STRING. **(B)** The top 100 DHX9-correlated genes in TCGA projects were acquired by GEPIA2. **(C)** The heatmap data of the 8 genes in the detailed cancer types. **(D)** An intersection analysis of the DHX9-binding and correlated genes. **(E)** KEGG pathway analysis of DHX9 binding and interacting genes. **(F)** The cnetplot function of the molecular function data in GO analysis.

To explore the molecular mechanism of DHX9 in tumorigenesis, an enrichment analysis was conducted to identify DHX9-binding proteins and DHX9-related genes. Our analysis revealed a total of 50 DHX9-binding proteins, supported by the STRING website (Figure 4A). Then we obtained the top 100 genes related to DHX9 expression in the TCGA database with the GEPIA2 tool. The expression level of DHX9 was positively correlated with HNRNPA2B1 (Heterogeneous Nuclear Ribonucleoprotein A2/B1), TARDBP (TAR DNA Binding Protein), HNRNPR (Heterogeneous Nuclear Ribonucleoprotein R), HNRNPC (Heterogeneous Nuclear Ribonucleoprotein C), KHDRBS1 (KH RNA Binding Domain Containing, Signal Transduction Associated 1), HNRNPK (Heterogeneous Nuclear Ribonucleoprotein K), NCL (Nucleolin) and DDX20 (DEAD-Box Helicase 20) (Figure 4B). In TIMER database, the corresponding heatmap showed that DHX9 was also positively correlated with these genes in most cancer types (Figure 4C). The Venn website was used to cross-analyze the related-gene group and the interaction-protein group, and 7 common members were found, namely, HNRNPR, KHDRBS1, NCL, DDX20, HNRNPA2B1, HNRNPK, and HNRNPC (Figure 4D). Enrichment analyses indicated that the overlapped genes were associated with “Spliceosome”, “RNA transport” and “mRNA surveillance pathway” (Figure 4E). According to GO enrichment analysis we found that these genes were associated with RNA metabolism and cellular processes, specifically mRNA 3'−UTR binding, poly−pyrimidine tract binding, poly(A) binding, single-stranded/poly(U) RNA binding, ribonucleoprotein granule, RNA/mRNA splicing, and other relevant functions (Figure 4F; Supplementary Figure S5).

### DHX9 was associated with immune infiltration and immune checkpoints

**FIGURE 5:**
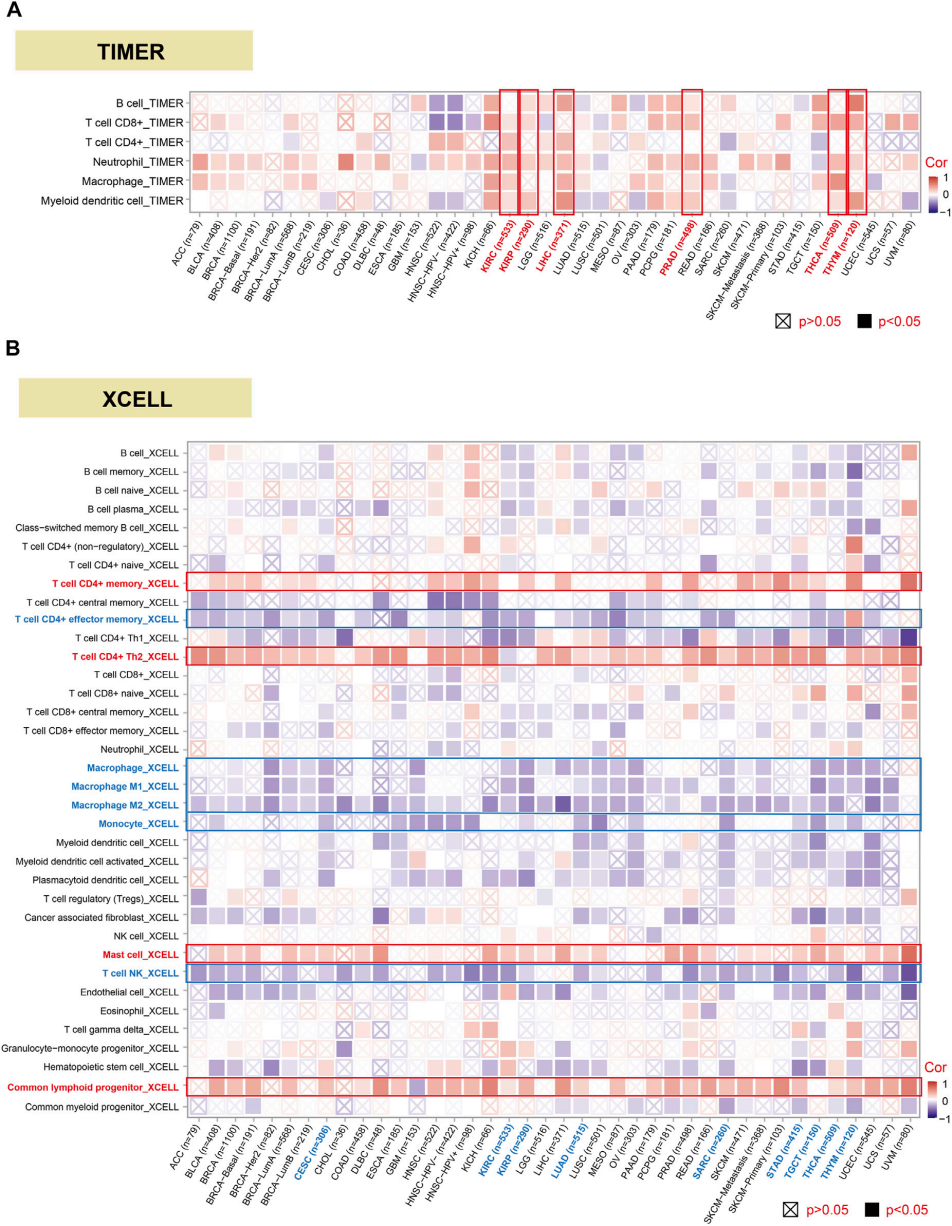
Correlation analysis between DHX9 expression and immune cell infiltration. **(A, B)** The potential correlation between the DHX9 expression and the infiltration level through TIMER and XCELL.

The investigation of the linkage among DHX9 expression, immune infiltration and immune checkpoints may provide a better understanding of the interaction between DHX9 and TME. Tumor-infiltrating immune cells are key components of TME and are closely related to the occurrence, progression, or metastasis of tumors (Fridman et al., 2011). Based on the TIMER database, we analyzed the correlation of DHX9 expression with immune infiltration levels in diverse cancer types. The expression of DHX9 was significantly associated with the abundance of infiltrating immune cells: B cells in 17 cancers, CD8^+^ T cells in 22 cancers, CD4^+^ T cells in 12 cancers, neutrophils in 31 cancers, macrophages and myeloid dendritic cells in 18 cancers (Figure 5A). In XCELL database, we found that DHX9 expression significant negatively correlated with immune cells in CESC, KIRC, KIRP, LUAD, SARC, STAD, TGCT, THCA, and THYM (Figure 5B). However, memory and Th2 CD4^+^ T cells, mast cells, and common lymphoid progenitor were most positively associated with the DHX9 in these different cancers, while effector memory CD4^+^ T cells and NK T cells, macrophages, and monocytes were negatively associated with the expression of DHX9 (Figure 5B).

**FIGURE 6:**
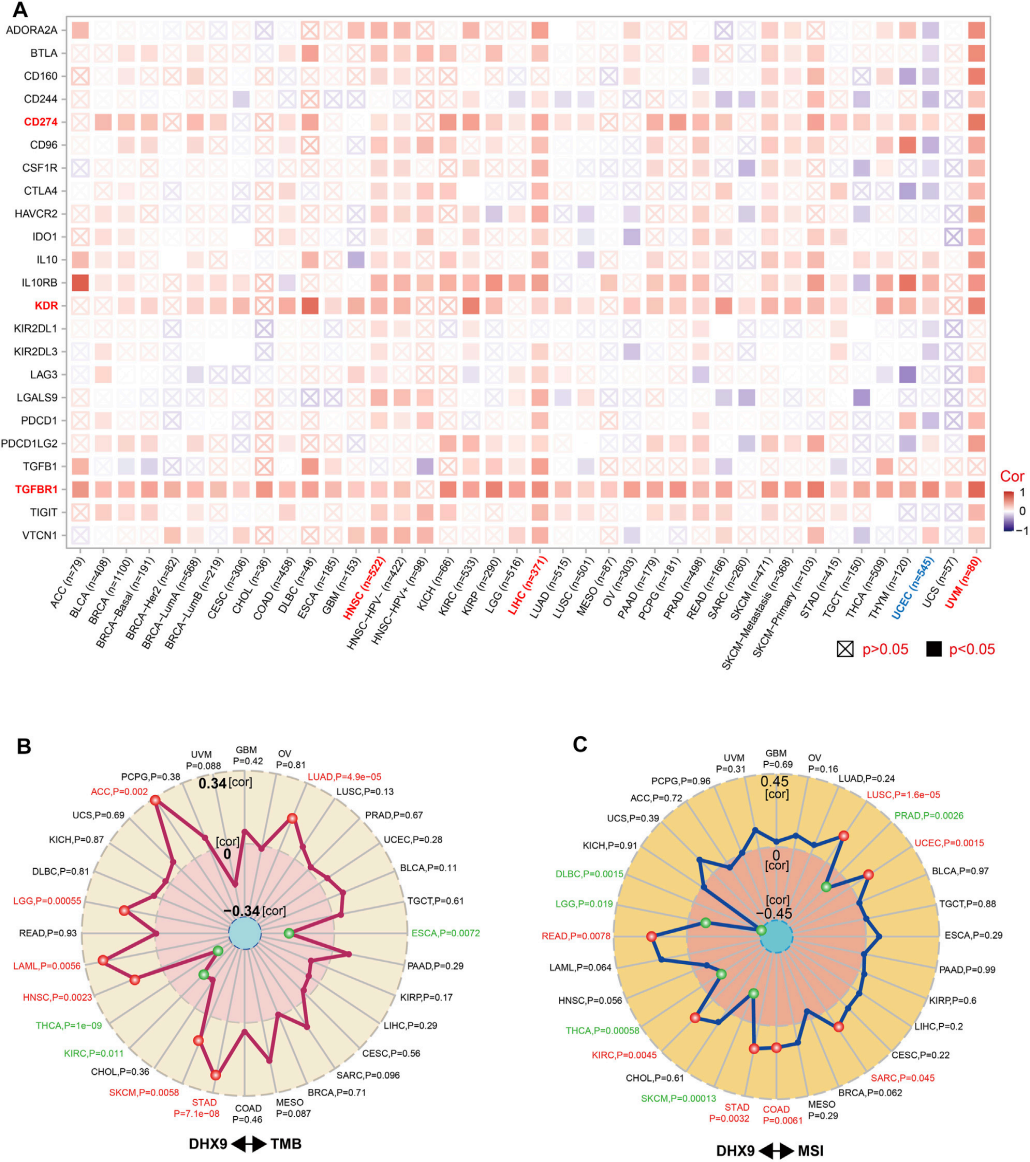
Correlation between DHX9 expression and immune checkpoint genes, and tumor mutational burden or microsatellite instability. **(A–C)** Based on the different tumors of TCGA, we explored the potential correlation between DHX9 expression and immune checkpoint genes **(A)**, tumor mutational burden (TMB) **(B)**, or microsatellite instability (MSI) **(C)**. The *p*-value is supplied and the partial correlation (cor) values of +0.34 and −0.34 in TMB and of +0.45 and −0.45 in MSI are marked.

Immunosurveillance is associated with the development and progression of cancer and affects the prognosis of patients with tumors (Dunn et al., 2002). Effective immune-checkpoint inhibitors that target CTLA-4 or the PD-1–PD-L1 axis, have been approved as targets for cancer treatment (Routy et al., 2018). We further investigated the relationship between DHX9 expression and two major types of immunomodulators (inhibitory and stimulatory immune checkpoints). Notably, the expression of DHX9 positively correlated with most immune-inhibitors and immune-stimulators in HNSC, LIHC, and UVM, while the expression of DHX9 negatively correlated with most immune modulators in UCEC (Figure 6A). In addition, DHX9 expression in most cancer types had an obvious positive correlation with several checkpoints, such as TGFBR1 (transforming growth factor-beta receptor 1), KDR (vascular endothelial growth factor receptor-2, VEGFR-2), and CD274 (PD-L1) (Figure 6A).

TMB and MSI are two emerging biomarkers associated with the immunotherapy response. In order to effectively guide the immunotherapy of tumors, we analyzed the correlation between DHX9 expression and TMB/MSI in all TCGA tumors. DHX9 expression was negatively correlated with TMB in THCA, KIRC and ESCA, but positively correlated in ACC, LGG, LAML, HNSC, SKCM, STAD and LUAD (Figure 6B). In addition, DHX9 expression was also negatively correlated with MSI in DLBC, LGG, THCA, SKCM and PRAD, but positively correlated with READ, KIRC, STAD, COAD, SARC, UCEC and LUSC (Figure 6C).

### DHX9 was highly expressed in cancer specimens

**FIGURE 7:**
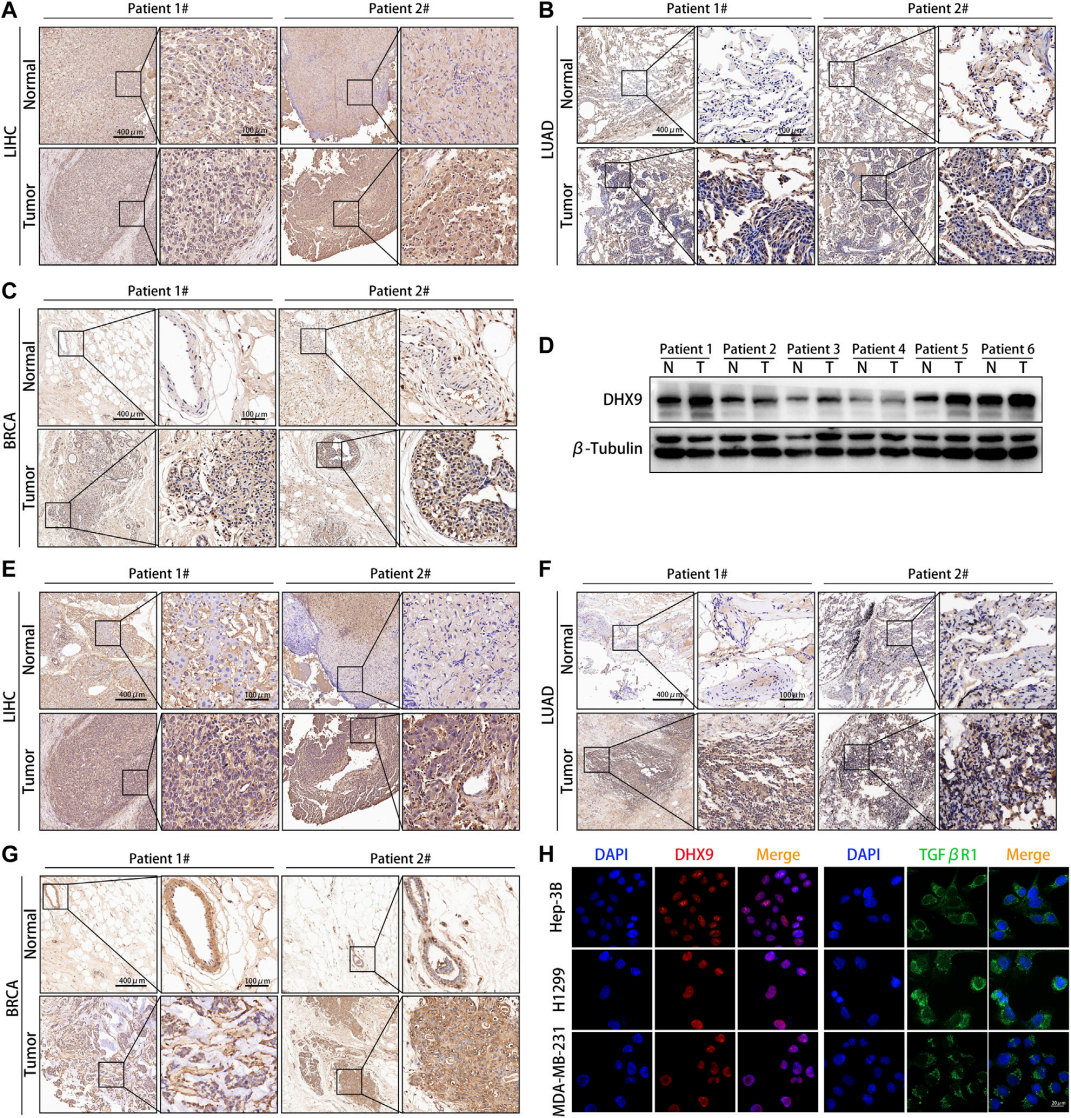
DHX9 was highly expressed in different cancer specimens. **(A–C)** Reprehensive image of the expression of DHX9 in tumor and adjacent normal tissues detected by IHC. **(D)** WB results showed the protein expression of DHX9 in 6 pairs of LIHC patients’ surgical specimens (T: tumor, N: normal tissue). **(E–G)** Reprehensive image of the expression of TGFβR1 in tumor and adjacent normal tissues detected by IHC. **(H)** IF images of the expression and localization of DHX9 and TGFβR1 in Hep-3B, H1299, and MDA-MB-231 cells. Scale bar, 20 μm.

We further explored the expression pattern of DHX9 in LIHC, LUAD and BRCA specimens by WB and IHC. The expression of the DHX9 was significantly higher in tumor tissues than in adjacent normal tissues in both LIHC, LUAD and BRAC (Figures 7A–C). Besides, in 6 pairs of LIHC samples, the expression level of DHX9 in tumor tissues was significantly upregulated (Figure 7D).

As the most significant immune checkpoint gene related to DHX9, we also examined the expression pattern of TGFβR1 in human cancers. Consistent with the DHX9 expression pattern, the expression level of TGFβR1 in tumor tissues was significantly higher than that in normal tissues (Figures 7E–G). Moreover, in patient 2 with higher expression of DHX9 (both in LIHC, LUAD and BRAC specimens), the expression level of TGFβR1 were upregulated accordingly, that further illustrates the positive correlation between DHX9 and TGFβR1 (Figures 7A–C,E–G). Furthermore, cell immunofluorescence showed that DHX9 was located in the nucleus, while TGFBR1 was located in the cytoplasm, indicating the spatial possibility of DHX9 regulating the expression of TGFβR1 (Figure 7H).

### DHX9 affected the proliferation and metastasis of cancer cells via regulating EMT

**FIGURE 8:**
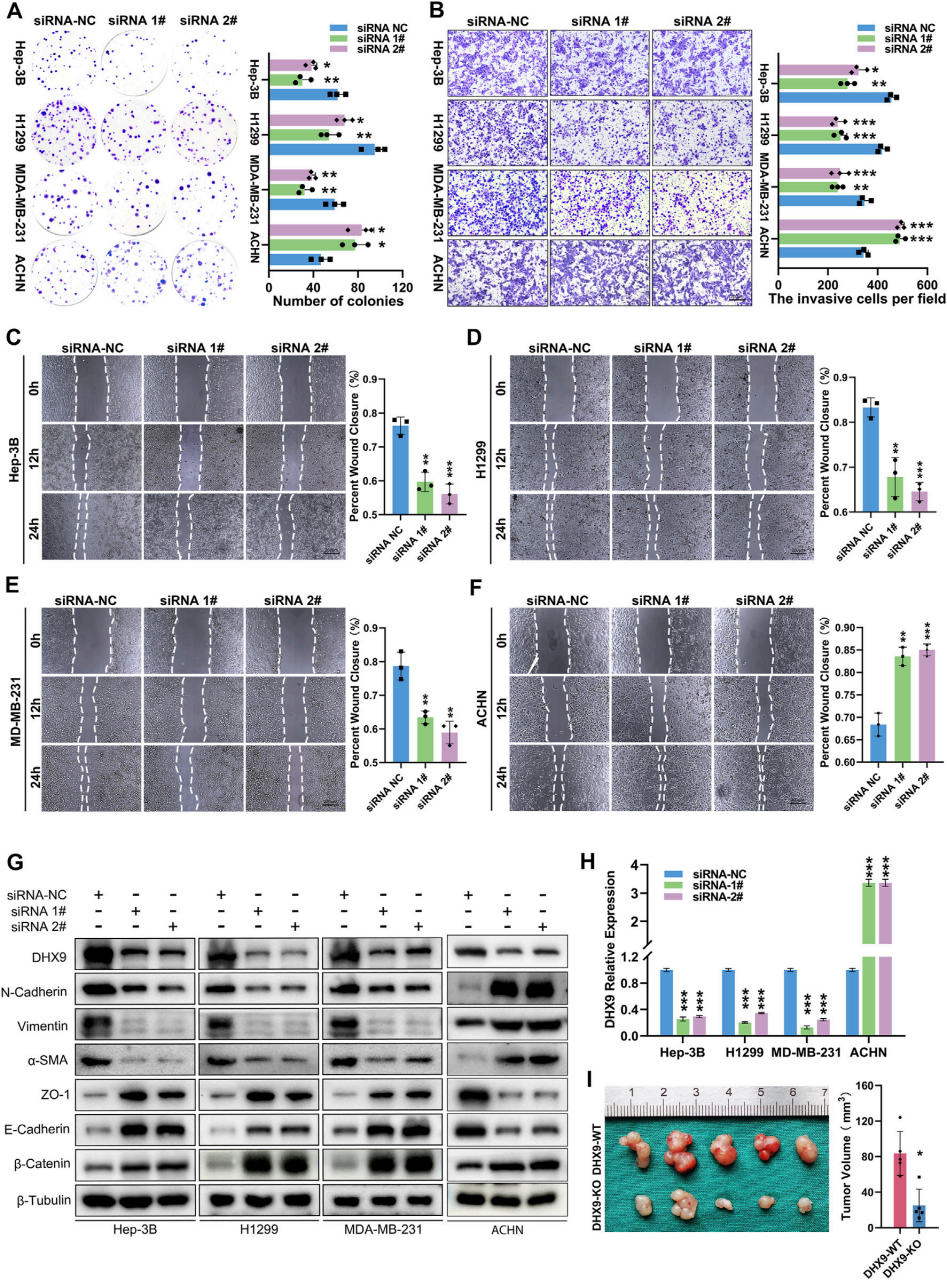
DHX9 mediates the proliferation, metastasis and EMT process of cancer cells. **(A)** Representative images and quantitative analysis of the colony formation assay for transfected Hep-3B/H1299/MDA-MB-231/ACHN cells. **(B)** Representative images and quantitative analysis of the transwell assay for transfected Hep-3B/H1299/MDA-MB-231/ACHN cells. **(C–E)** Representative images and quantitative analysis of the Wound healing assay for transfected Hep-3B/H1299/MDA-MB-231/ACHN cells. **(F)** Western blot visualization of altered expression of EMT related molecules after knockdown DHX9 in Hep-3B, H1299, MDA-MB-231and ACHN cell lines. **(G)** The efficiency of silencing DHX9 was indicated by RT-qPCR in Hep-3B, H1299, MDA-MB-231, and ACHN cell lines. **(H, I)** Representative images and quantitative analysis of tumors *in vivo* experiments. * *p* < 0.05; ** *p* < 0.01; *** *p* < 0.001.

After validated the expression pattern of DHX9 in human cancer, we also explored its function in cancer cells. The DHX9 knockdown efficiency in Hep-3B, H1299, MDA-MB-231 and ACHN cells was validated by RT-qPCR and western blot (Figures 8G, H). Colony formation assays revealed that the silencing of DHX9 suppressed cell proliferation of Hep-3B/SK-Hep-1, H1299/PC9 and MDA-MB-231/MCF7 cells, but promoted the proliferation of ACHN/OS-RC-2 cells (Figure 8A; Supplementary Figure S6A). Wound healing assays and transwell assays revealed that the inhibition of DHX9 significantly suppressed the metastasis ability of Hep-3B/SK-Hep-1, H1299/PC9 and MDA-MB-231/MCF7 cells, but promoted the metastasis of ACHN/OS-RC-2 cells (Figures 8B–F; Supplementary Figure S6B-F). Previous studies indicated that DHX9 might affect the metastasis of bladder cancer by promoting epithelial-mesenchymal transition (EMT) (Yan et al., 2019). Therefore, we detected the expression level of EMT related molecules after DHX9 knockdown. Results showed that the epithelial markers (ZO-1, E-Cadherin, *ß*-Catenin) were increased and mesenchymal markers (N-Cadherin, Vimentin, *a*-SMA) were downregulated after DHX9 knockdown in liver cancer, lung cancer and breast cancer cells, indicating that DHX9 promoted EMT in these cancers, however, the opposite results were detected in renal cell carcinoma cells (Figure 8G).

To further confirm the role of DHX9 in EMT, we generated a DHX9-knockout plasmid to deplete DHX9 expression, which had a greater efficacy of DHX9-knockout than shRNA-mediated silencing. The knockout of DHX9 had the same effects on EMT as DHX9 knockdown in liver cancer, lung cancer, breast cancer, and renal cell carcinoma cells (Supplementary Figure S7A). Furthermore, subcutaneous xenograft tumor model was established and revealed that the knockout of DHX9 markedly suppressed the growth of Hep-3B cells (Figure 8I; Supplementary Figure S7B).

These results suggested that DHX9 could promote the proliferation and metastasis of liver, lung and breast cancers but suppress those of renal cell carcinoma. Its pathogenic role depended on the regulation of EMT in cancers.

## Discussion

Given the complex tumor pathogenesis and unsatisfactory therapeutic effect, preventing the occurrence of the tumor, improving the diagnosis level, and finding new therapeutic targets have always been the common pursuit of scientific researchers and clinicians (Morata and Calleja, 2020). DHX9, as an NTP-dependent RNA helicase, plays an important functional role in the occurrence and development of tumors (Hong et al., 2018). Our study demonstrates that DHX9 as a probable prognostic biomarker for various cancer types and it was associated with immune infiltration. Furthermore, *in vivo* and *in vitro* experiments revealed that DHX9 could affect the proliferation, metastasis of liver, lung, breast and renal cancer cells *via* regulating EMT.

DHX9 has been implicated to be involved in tumorigenesis of various cancers. DHX9 mediates the downregulation of circDCUN1D4, which is more common in clinically metastatic lymph nodes, resulting in a poor prognosis of cancer patients (Liang et al., 2021). And increased DHX9 blocks the DNA repair function of BRAC and leads to cancer development (Schlegel et al., 2003). In addition, as a downstream gene of SOX4, DHX9 activates the Wnt/β-catenin signaling pathway then promotes tumor metastasis and drug resistance (Jiang et al., 2007; Gupta et al., 2010; Thiago et al., 2010; Lai et al., 2011). Moreover, it may act as a negative regulator of tumorigenesis in distinct tumor types. Previous studies indicated that the knockdown of DHX9 enhanced the proliferation and migration of thyroid cancer and renal cancer cells. (Gong et al., 2021; Liu et al., 2021). In prostate cancer (PC), depletion of DHX9 in PC cells suppressed androgen-induced cell proliferation and migration (Chellini et al., 2022). DHX9 expression was regulated by RNF168-mediated ubiquitination. The knockdown of RNF168 could lead to reduced recruitment of DHX9 to the R-loop, impeding the resolution of the R loops. The accumulation of R-loops in tumor cells contributed to double-strand breaks, senescence, and subsequent cell death (Patel et al., 2021). However, the pan-cancer expression profile and prognostic significance of DHX9 remain uncharacterized. This present study demonstrated that DHX9 was overexpressed in most tumors, but low expression of DHX9 was shown in KICH, KIRC, KIRP, and THCA tumors. In LUAD, LIHC, BRCA, and PRAD, upregulated DHX9 indicated poor survival. The expression of the DHX9 was significantly higher in tumor tissues than in adjacent normal tissues in both LIHC, LUAD and BRAC. Moreover, our results reveal that DHX9 can facilitate cellular proliferation and metastasis in Hep-3B, H1299 and MDA-MB-231 cells *via* promoting EMT process; however this phenomenon is not observed in renal cancer cells, where DHX9 expression appears to reciprocally suppress the cancerous and EMT pathway, therefore, our results are consistent the previous findings (Gong et al., 2021). These results indicated that DHX9 was a prognostic biomarker in most cancers and it might be a target for cancer treatment.

As one of the most widely studied post-translation modification, protein phosphorylation plays an important role in the cellular processes. Recent studies have revealed the importance of abnormal protein phosphorylation in cancer progression (Roberts and Der, 2007; Singh et al., 2017). Studies have shown that some post-translation modifications of DHX9 occur in cancer or drug-resistant cells and may be therapeutic targets for cancer (Cao et al., 2017). In this study, we used CPTAC to analyze data from 6 tumor types and found that the DHX9 phosphorylation level of site S449 was significantly lower only in ccRCC compared with normal tissues. Intriguingly, the S688 locus was only expressed in ccRCC with an obvious reduction. These results suggested that S449 and S688 were specific phosphorylation sites of ccRCC and might become new targets for tumor prediction.

Predicting the efficacy of immunotherapy for cancer patients is now becoming a clinical demand (Gajewski et al., 2013). The presence of tumor-infiltrating immune cells is critical to the tumor microenvironment, thus, comprehensive assessment of immune infiltration status is important for selecting the correct individualized immunotherapy strategy (Zhang and Zhang, 2020; Chen et al., 2021). The potential relationship between DHX9 and tumor immune microenvironment remains insufficiently explored. Our present study reveals a significant association between DHX9 expression levels and the infiltration of various immune cells in different cancers, including CD4^+^ T cells, CD8^+^ T cells, neutrophils, B cells, DCs and macrophages. Besides, our analyses unveiled a remarkable correlation between DHX9 expression levels and a group of immune checkpoint genes that contain immunosuppressive and immunostimulatory genes in HNSC, LIHC, UVM, and UCEC. DHX9 expression was significantly associated with expression of t immunosuppressive and immunostimulatory molecules especially TGFβR1, which was further validated by IHC. Furthermore, the results of cell IF indicated the spatial possibility of DHX9 regulating the expression of TGFβR1, which required additional investigation.

TMB can predict immunotherapy efficacy for multiple cancer types. In most cases, high TMB indicates favorable response to immunotherapy (Samstein et al., 2019). Our findings indicated a significant positive association between DHX9 expression and TMB values in patients with LGG, LUAD, HNSC, and STAD, potentially predicting the response to immunotherapy. MSI is a biomarker of immune checkpoint inhibitors (ICIs) response (Samstein et al., 2019). Tumors that are sensitive to immunotherapy are those with high MSI value (Baretti and Le, 2018). In the present study, we found that there existed two types of tumors: DHX9 was highly expressed and had a positive correlation with MSI, such as COAD, LUSC, READ, STAD, and UCEC; and DHX9 was lowly expressed but had a negative correlation with MSI, such as THCA. Therefore, DHX9 expression shared similar trends with both TMB and MSI values, which suggested that DHX9 was a promising biomarker to predict the efficacy of immunotherapy in cancers.

Collectively, our study comprehensively characterized the expression pattern and prognostic role of DHX9 in pan-cancer. DHX9 was a potential biomarker to predict immunotherapy efficacy and a target for cancer treatment.

## Data Availability

The data that support the findings of this study are available from the corresponding author upon reasonable request.

## Supplementary material

SUPPLEMENTARY FIGURE S1 Structural characteristics and expression pattern of DHX9 in different species, tissues or cells. **(A)** Genomic location of human DHX9. **(B)** Conserved domains of DHX9 protein among different species. **(C)** DHX9 phylogenetic tree across different species (get by NCBI). **(D, E)** DHX9 expression in different cells, plasma, and tissues through the consensus dataset of HPA, GTEx, and FANTOM5. **(E)** DHX9 expression in different blood cells through the consensus dataset of HPA, Monaco, and Schmiedel.

SUPPLEMENTARY FIGURE S2 Expression level of the DHX9 in different tumors and pathological stages. **(A)** The mRNA expression of the DHX9 between ACC, LAML, OV, SARC, SKCM, TGCT, UCS of TCGA project, and the corresponding normal tissues of GTEx databases. (B-E) Expression levels of the DHX9 gene by different pathological stages of cancers.

SUPPLEMENTARY FIGURE S3 Several compared analyses of the DHX9 expression between normal and tumor tissues. **(A)** breast cancer; **(B)** lung cancer; **(C)** sarcoma; **(D)** colorectal cancer; **(E)** brain cancer. Based on the Oncomine database.

SUPPLEMENTARY FIGURE S4 The protein expression level of DHX9 in different cancers. The immunohistochemical images showed that high DHX9 expression was detected in BRCA **(A)**, COAD **(B)**, and LUAD **(C)**, while low expression in KIRC **(D)**. Based on the The Human Protein Atlas database(https://www.proteinatlas.org).

SUPPLEMENTARY FIGURE S5 KEGG pathway and GO analysis of DHX9-related genes. Based on the DHX9-binding and interacted genes, KEGG enrichment analyses were performed for the biological process **(A)**, cellular component **(D)**, and molecular function **(E)**. GO enrichment analyses were displayed for the biological process **(B)** and cellular component **(C)**.

SUPPLEMENTARY FIGURE S6 DHX9 mediates the proliferation and metastasis of cancer cells. **(A)** Representative images and quantitative analysis of the colony formation assay for transfected SK-Hep-1/PC9/MCF7/OS-RC-2 cells. **(B)** Representative images and quantitative analysis of the transwell assay for transfected SK-Hep-1/PC9/MCF7/OS-RC-2 cells. **(C-F)** Representative images and quantitative analysis of the wound healing assay for transfected SK-Hep-1/PC9/MCF7/OS-RC-2 cells. * P < 0.05; ** P < 0.01; *** P < 0.001.

SUPPLEMENTARY FIGURE S7 DHX9 promotes the metastasis of HCC cells via EMT. **(A)** Western blot visualization of altered expression of EMT related molecules after knockout DHX9 in SK-Hep-1/PC9/MCF7/OS-RC-2 cell lines. **(B)** Representative images of nude mice bearing tumor in vivo experiments.
