# Supplementary figures and images for: A pan-cancer analysis of the expression and molecular mechanism of DHX9 in human cancers
Source: Front Pharmacol. 2023 May 4;14:1153067. doi: 10.3389/fphar.2023.1153067 (PMC10192771; doi:10.3389/fphar.2023.1153067)

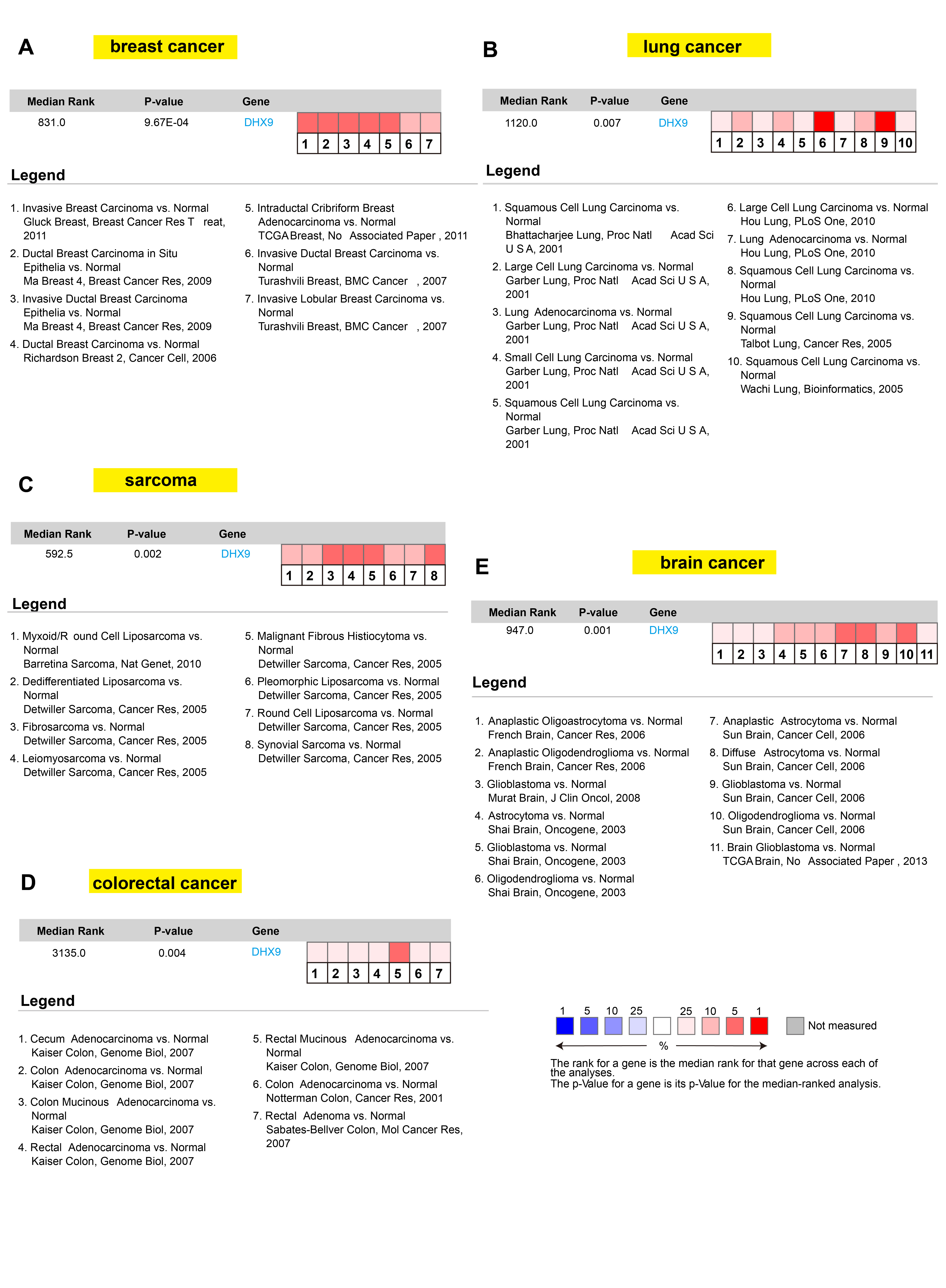

Supplement: Supplementary file 1 [file Image3.JPEG]

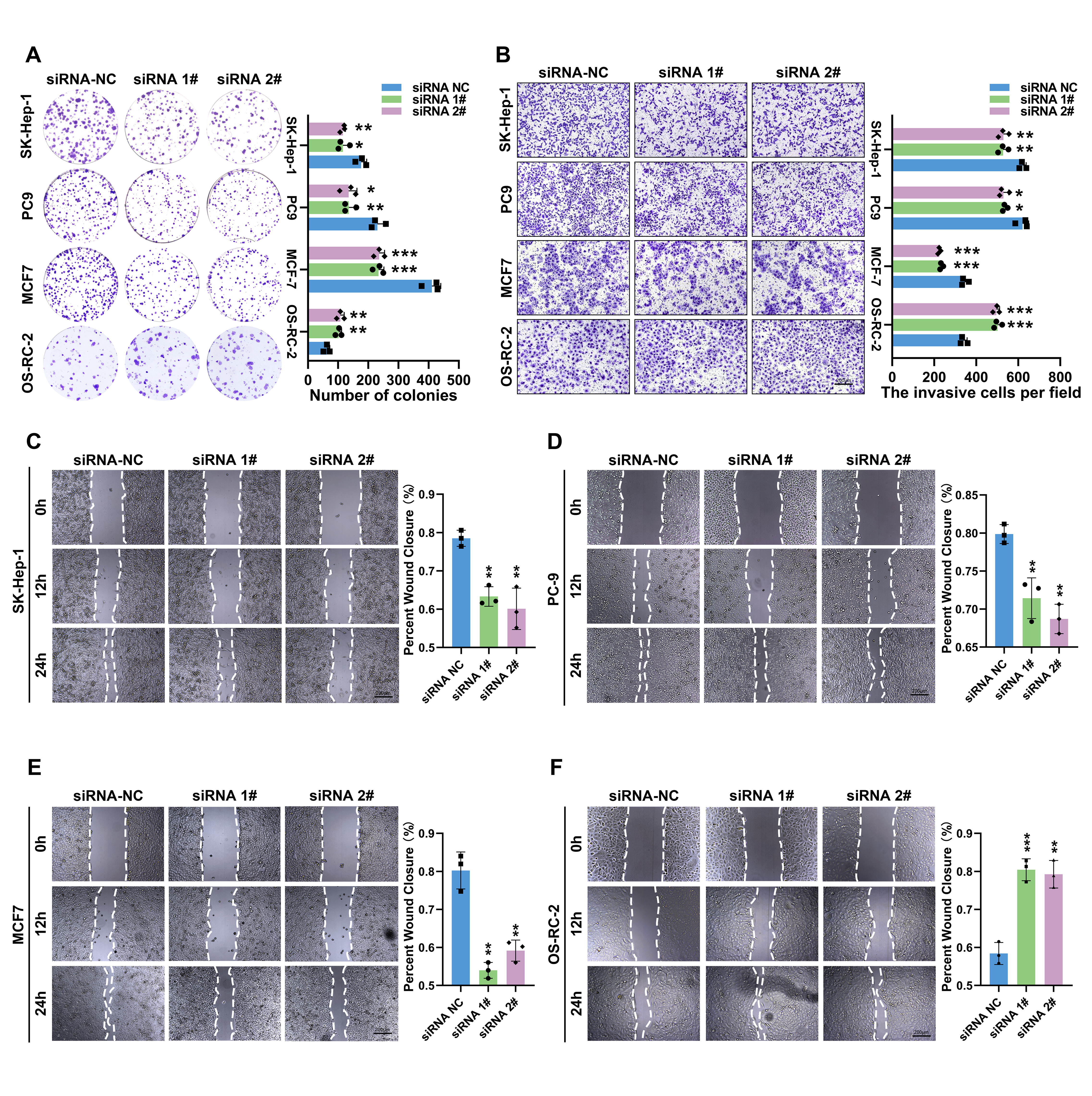

Supplement: Supplementary file 2 [file Image6.TIF]

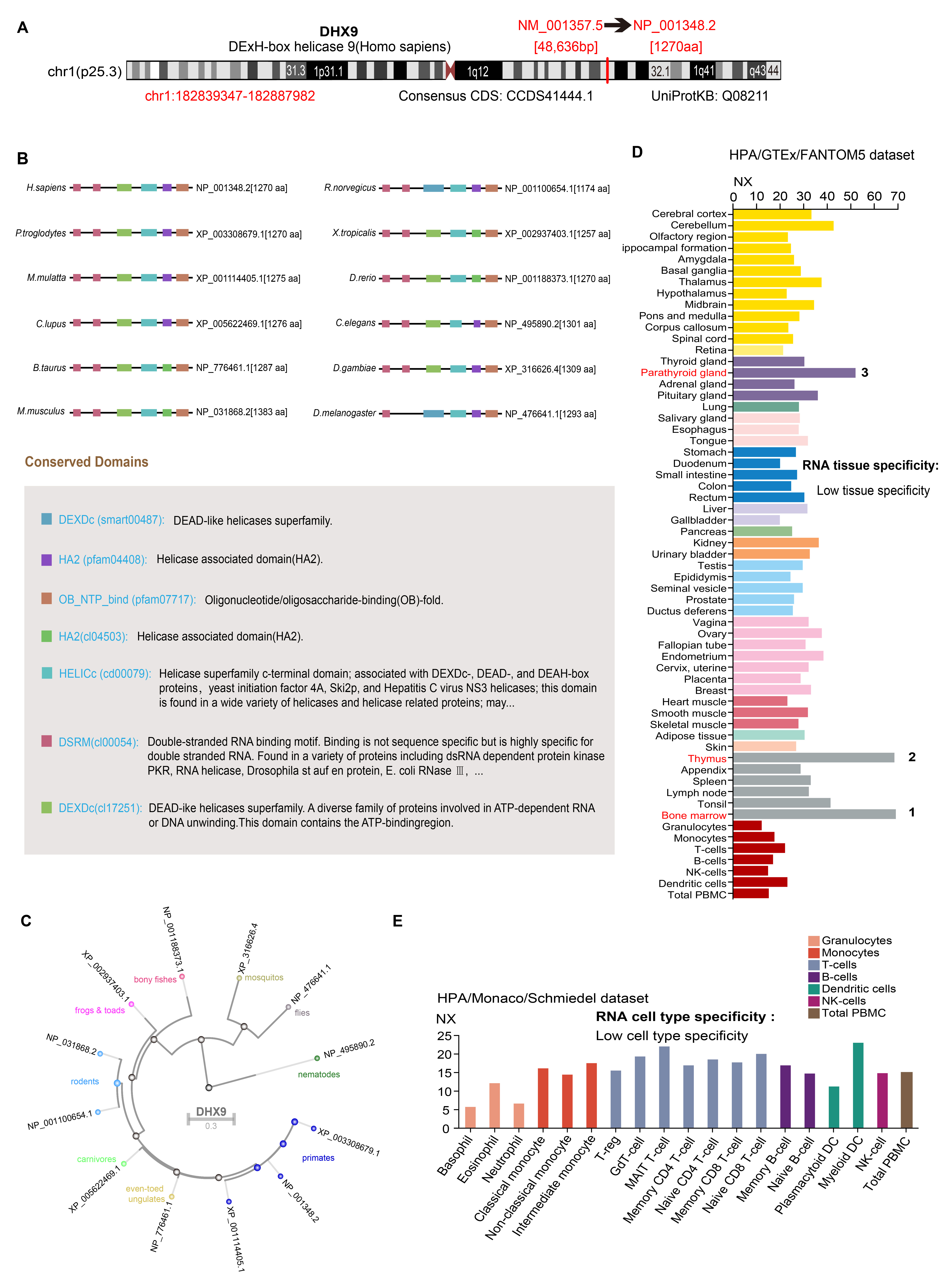

Supplement: Supplementary file 3 [file Image1.JPEG]

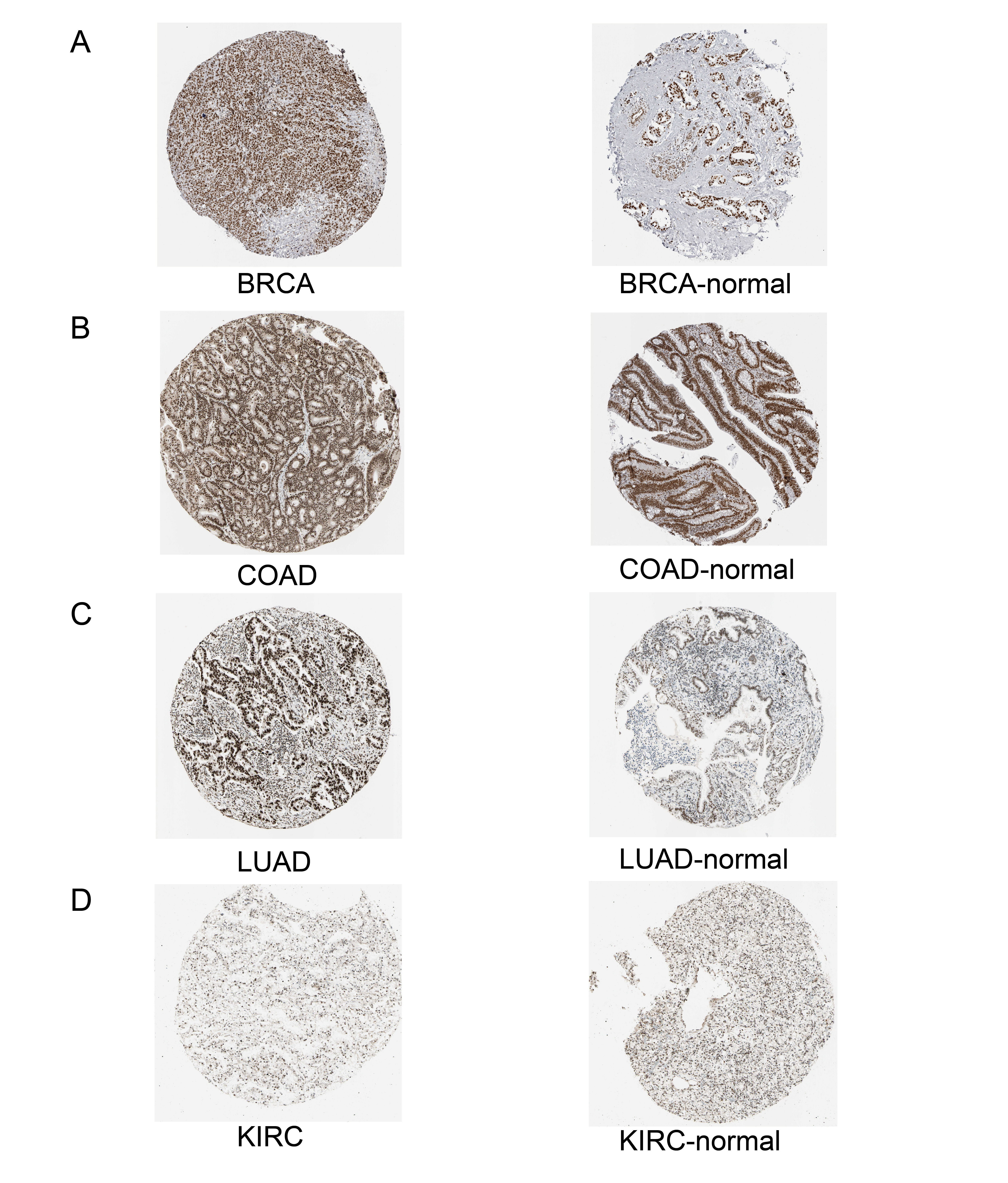

Supplement: Supplementary file 4 [file Image4.JPEG]

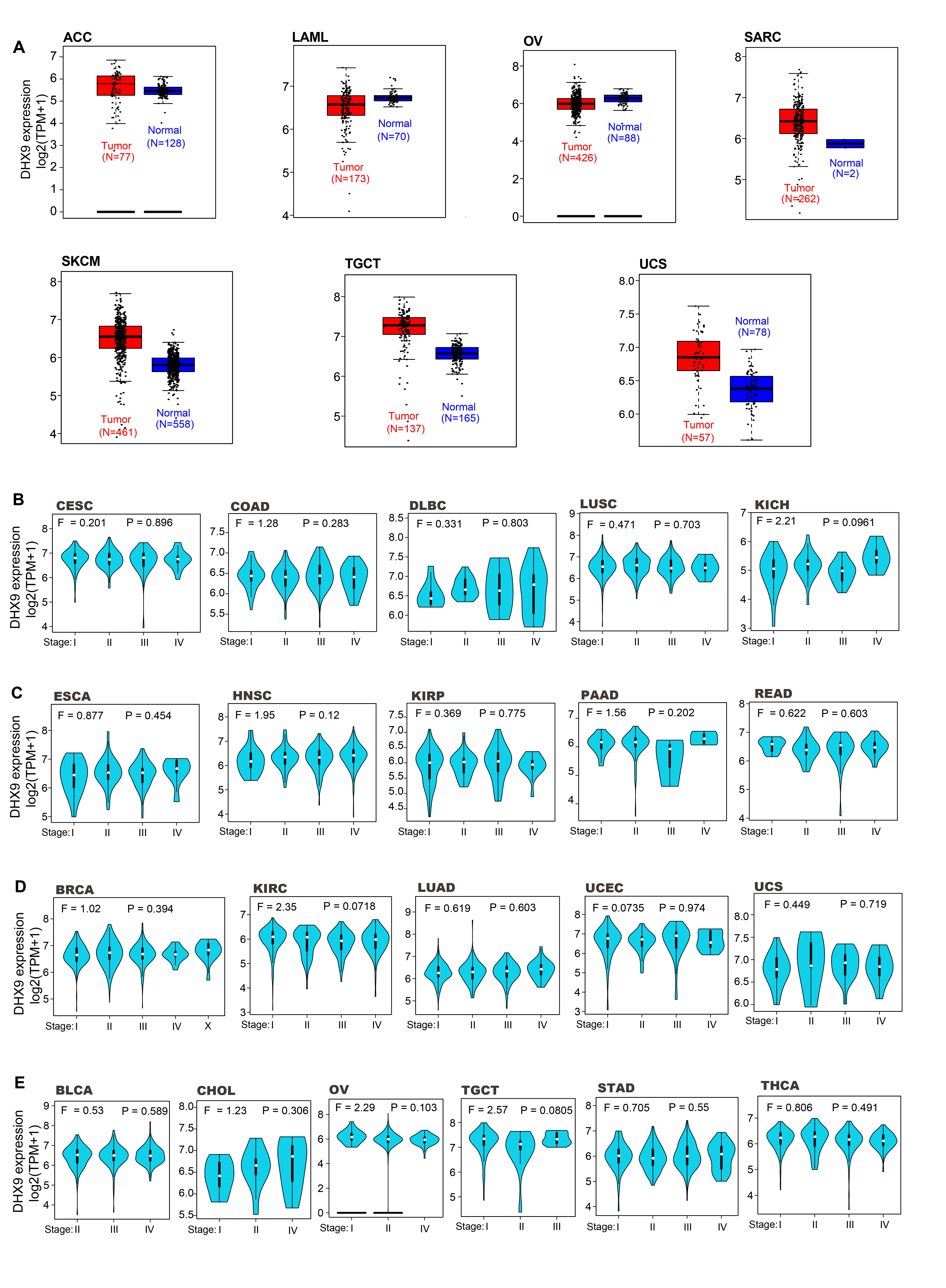

Supplement: Supplementary file 5 [file Image2.JPEG]

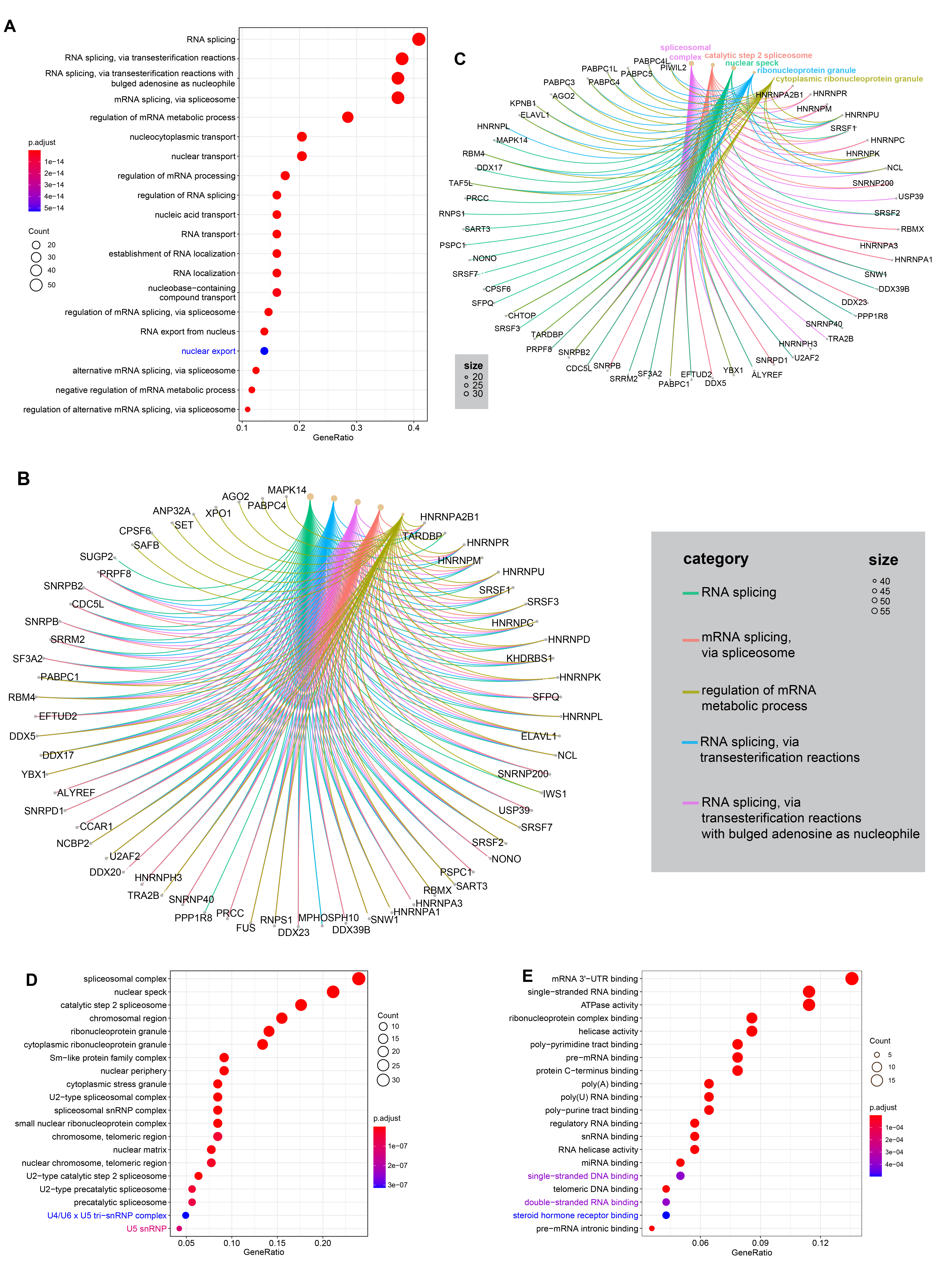

Supplement: Supplementary file 6 [file Image5.JPEG]
